# Genome Sequence of a Clinical *Klebsiella pneumoniae* Sequence Type 6 Strain

**DOI:** 10.1128/genomeA.01311-15

**Published:** 2015-11-12

**Authors:** Cyril Guilhen, Agnès Iltis, Christiane Forestier, Damien Balestrino

**Affiliations:** a Laboratoire Microorganismes: Génome et Environnement, UMR CNRS 6023, Université d'Auvergne, Clermont-Ferrand, France; b Genostar, Montbonnot Saint-Martin, France

## Abstract

We report here the genome sequence of *Klebsiella pneumoniae* CH1034, a sequence type 6 (ST6) strain isolated in 2012 from a central venous catheter of a hospitalized patient.

## GENOME ANNOUNCEMENT

*Klebsiella pneumoniae* is an important Gram-negative opportunistic pathogen frequently associated with nosocomial infections, including urinary tract infections, pneumonia, infections of surgical wounds, bacteremia, and septicemia (1, 2). In contrast to many other bacterial pathogens, *K. pneumoniae* is ubiquitous in nature, and nonclinical isolates from the environment were found to be as virulent as clinical strains (3, 4).

Here, we report the unscaffolded whole-genome shotgun (WGS) sequence of the *K. pneumoniae* strain CH1034 isolated from a central venous catheter of a patient hospitalized in the teaching public hospital of Clermont-Ferrand, France, in 2012. The strain belongs to multilocus sequence type 6 (ST6) with a K7 capsular serotype (5, 6) and is resistant to ampicillin and ticarcillin; however, it was found to be susceptible to other β-lactams, aminoglycosides, quinolones, fluoroquinolones, sulfamethoxazole-trimethoprim (Bactrim), nitrofurans, and third-generation cephalosporins.

The genomic DNA from CH1034 was sequenced by next-generation sequencing using an Illumina HiSeq 2000 instrument with 100-bp paired-end reads and 2,000× coverage. After trimming, a total of 109,160,225 reads with a mean read length of 93.42 bp were *de novo* assembled using clc_assembler from the CLC Genomics Workbench analysis package with standard parameters. The 46 resulting contigs, ranging from 567 bp to 412,668 bp, were ordered by genome position and orientation using the previously published *K. pneumoniae* NTUH-K2044 genome as a reference with Mauve Contig Mover (7); these were subsequently imported into the MicroScope database system. The genome was annotated automatically within the MicroScope platform and manually visualized using the Magnifying Genomes (MaGe) Web interface (8, 9). Annotation results relative to BLAST similarities, Clusters of Orthologous Groups (COG) assignations, enzymatic function prediction (PRIAM software), TMHMM, PsortB and SignalP predictions, and synteny conservation (Syntonizer software) are available in the MicroScope annotation platform (http://www.genoscope.cns.fr/agc/microscope).

The assembled genome size of isolate CH1034 was 5,271,927 bp in length, with a G+C composition of 57.4%. Genome annotation resulted in 5,248 genomic objects, including 5,114 predicted coding sequences (CDSs), 15 pseudogenes, 5 rRNAs (3 5S rRNAs, 1 16S rRNA, and 1 23S rRNA), 70 tRNAs, and 44 miscellaneous RNAs (miscRNAs). The annotation results showed that 1,245 CDSs were annotated with at least 1 EC number, corresponding to 138 KEGG maps, with at least one reaction catalyzed in *K. pneumoniae* CH1034. Moreover, 83.77% of the CDSs are classified in at least one COG group (4,297/5,129 CDSs), of which 8.75% (376/4,297 CDSs) are classified in the unknown function group. Eleven putative transposase-encoding genes were detected in the genome.

A comparison with the genome sequences available in the MicroB (version 022015; Genostar) and nr databases showed that a region of 16.4 kb (CDSs *CH1034_250036* to *CH1034_250050*) was found in only 3 other bacterial genomes: *K. pneumoniae* subsp. *pneumoniae* PittNDM01, *Enterobacter hormaechei* isolate 05545, and *Escherichia coli* ECOR31. Among the 15 CDSs that were annotated, 5 encoded potentially proteins with enzymatic functions (helicase, primase, peptidase, and patatin), and 10 encoded potentially proteins with unknown function.

### Nucleotide sequence accession numbers.

This whole-genome shotgun project has been deposited at ENA/EMBL-EBI under the accession no. PRJEB9899 and contig accession numbers CXPD01000001 to CXPD01000046.
